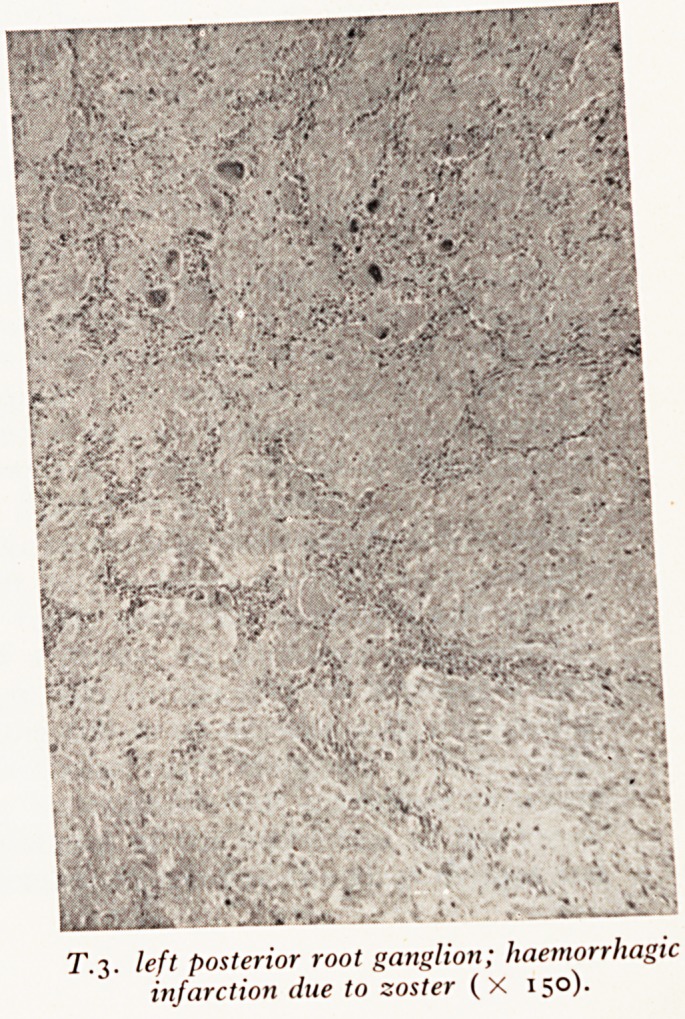# Herpes Zoster with Chicken-Pox and Lymphosarcoma

**Published:** 1959-01

**Authors:** O. C. Lloyd


### HERPES ZOSTER WITH CHICKEN-POX AND LYMPHOSARCOMA

*A Clinical Pathological Conference of the University of Bristol Medical School* (
*20th May* 1958.

CHAIRMAN: DR. O. C. LLOYD

*Dr. Warin:* This patient was an old man of 80, with no significant past history (
family history.

On 26th July, 1957 he developed pain in the left side of the chest, followed by
rash on the upper part of the chest and one day later he developed a generaliz
rash which continued to erupt for two to three days. He was admitted to the Brist
Royal Infirmary. Examination showed typical herpes zoster with some gangreno
areas in the third and fourth thoracic dermatomes on the upper chest running roun
to the back; also a widespread eruption consisting of vesicles, many with haemor
hages into them, over the trunk, limbs, hands and feet. He was very ill, with a hi
temperature.

After two days he started to vomit, was restless and his abdomen was distende
Mr. Monks saw him and decided there was a subacute intestinal obstruction. H
was, however, too ill for operation and we decided to watch and give intraveno
fluids. After three days his general condition had improved and Mr. Monks operat
and found an obstructed bowel. The patient died two days later.

There are one or two points I would like to mention, firstly the actual appearan
of the rash was very like smallpox. He had vesicles characteristic of smallpox in ty
and distribution, and severe toxaemia. However I was reassured by the band on t
left side of the chest—clearly a herpes zoster.

The viruses of herpes zoster and of chicken-pox are closely related and there is
doubt that contacts of herpes zoster may develop chicken-pox. This was demonstrat
in this case by the fact that our Registrar developed chicken-pox two weeks af
this patient was admitted, and had a very severe, haemorrhagic eruption. Our wa
staff nurse had not had chicken-pox and she informed us that she was three mont
pregnant—what should we do? I rang up Professor Perry who decided that the be
thing was to give her gamma globulin. I would like to know whether you think th
is a real risk of chicken-pox in early pregnancy and whether gamma globulin wou
prevent it.

The other point I want to make is that a few aberrant vesicles in cases of herp
zoster are quite common, but this very widespread rash with herpes zoster is r
and there is usually some underlying debilitating state when it does occur. Dr. M
Hewitt (1954) recorded four cases in our own *Journal*, and of these one had Hodgki
disease and another carcinoma.

Plates VII, VIII and IX show the patient from the back, front and side views.

*Mr. Monks:* Dr. Warin asked me to see this man on the 2nd August, two da
after admission, because he was vomiting and his abdomen was distended. Wh
we examined him he was dehydrated, his abdomen was grossly distended and we co
feel loops of bowel, and could actually feel them contracting. He also had loud a
tinkling bowel sounds. It was difficult to decide whether the man had colic or not,
was rather disorientated.

Operative treatment was unjustified at this stage; we did not know what was t
cause of this obstruction. He was therefore given an enema and passed a considera
amount of flatus. He had a nasal tube and we aspirated moderate amounts of sm
bowel contents. We continued in this manner, giving frequent enemas, sucks o
and an intravenous drip until the 6th August. By this time though the abdomen v becoming progressively more distended he was maintaining his general condition fairly well, and if anything it had improved; and by the 6th August we felt that this old man was grimly holding on to life. We had in mind the possibility of an underlying malignant disease, probably carcinoma of the colon, and thought that we should do a colostomy.

On the 6th August we opened the abdomen and found he had a grossly distended small and large bowel and there was serous fluid in the peritoneal cavity; in his mesentery there were some very large discrete glands (up to about 2 in. diameter). We could find no definite point of obstruction of the bowel so we deflated it, and closed the abdomen. We thought it was some kind of lymphosarcoma or other tumour causing ileus by interfering with the innervation of the bowel.

Following this he went gradually downhill and died about 48 hours later.

Dr. Doyle showed X-Ray photographs: Plate X is an antero-posterior film with the patient lying supine; there is moderate distension in numerous small bowel loops, but with considerable air and faecal content in the ascending, transverse and descending colon. The films did not show the whole of the bowel but we suggested it was most likely to be an ileus.

*Dr. McConnell:* What is the significance of increased bowel sounds?

*Mr. Monks:* The bowel sounds are a sign to be taken into consideration, when you have distension. With bowel sounds loud and tinkling there is more likelihood of it being obstruction than ileus.

*Dr. Lloyd:* In what circumstances do you get complete absence of bowel sounds?

*Mr. Monks:* It depends how long you listen. It is necessary to listen for quite some time. Perforated duodenal ulcers are characterized by absent bowel sounds when you see them fairly early. After a time the sounds return. In an internal strangulation which is the most severe form of intestinal obstruction, there may be no sounds if the obstruction is very high. When bowel sounds are absent post-operatively it helps to distinguish between peritonitis and post operative obstruction due for instance to a bit of bowel caught into a hole in the mesentery. The presence or absence of bowel sounds is useful at times to distinguish between the two. During the war this was a useful sign of intra-abdominal injury.

*Dr. Lloyd:* Would it be true to say that the presence of bowel sounds means that surgical interference is more likely to be effective than it would be in the absence of sounds?

*Mr. Monks:* In this type of case, yes. They indicate that there is gas and fluid in the small bowel and the small bowel is contracting. This must be taken into consideration with the other findings.

*Question:* How is it possible completely to exclude smallpox in a case like this?

*Dr. Warin:* In this case it was easy because of the area of herpes zoster and the pain and the mode of onset. But of course there is a very real difficulty in differentiating between a chicken-pox and a smallpox, particularly a smallpox modified by vaccination, and I think it can at times be impossible. The rash of smallpox is more marked on the extremities and shotty vesicles and pustules occur on the palms and soles. There is quite a lot made of whether the vesicles are unilocular or multiocular but I do not think it very important. The virus of smallpox can be demonstrated with a fair degree of certainty by a direct smear of the vesicular fluid.

*Question:* Were there any vesicles on the mucous membrane?

*Dr. Warin:* Yes, they were to be seen in the buccal mucosa.

*Dr. Lloyd:* The rash in this case was worst on the trunk, but would it not be true to say that generally speaking his hands had been free?

*Dr. Warin:* No. He had quite a lot on his hands and feet, maybe they had faded when you saw him, but he had these typical shotty vesicles that one could demonstrate quite easily.

*Question:* Can they be seen in the bladder in herpes zoster?

*Dr. Warin:* I do not know of any report.

*Dr. Lloyd:* I think at this stage it would be a good thing for me to tell you ab the post mortem findings:

The body was slightly wasted and there was still some distension of the abdom The rash was as has been described to you already, both the haemorrhagic chicken- rash and also the confluent zoster rash, particularly in the distribution of the th left thoracic dermatome. This was very much infected posteriorly so that there a great deal of pus being formed. I think the secondary infection accounted a gr deal for the toxic symptoms from which he was suffering. There was a little fibrin peritonitis, particularly in the lower half of the abdomen, but it was not a very p nounced finding.

The myocardium showed some scattered areas of fibrosis but this was not pa cularly significant. There was hypostatic compression collapse of the lungs affect particularly the right lower lobe, where there was acute bronchiolitis. The spl was greatly enlarged, rather soft and adherent to the diaphragm. The lymph no showed moderate enlargement of all groups with the exception of the mediasti paratracheal and inguinal nodes. The enlargement was greatest in the case of mes teric nodes. The left subclavian nodes were much larger than the right, possi because of the suppurating zoster rash. The stomach was severely dilated and mucosa dotted all over with small acute erosions. The small intestine was gre dilated (up to 12 cm. circumference, but in the terminal ileum only 8 cm. circu ference). There was a polypoid submucosal lipoma (2 cm. diameter) at the junct of the jejunum and ileum but it was not causing obstruction. The colon was moderately dilated. There was evidence of the small intestine having been ope and neatly sewn up again in the manner which has been described. The prostate been partly removed at a previous operation; there was slight nodular enlargem of the right lobe, not causing any obstruction.

Dr. Lloyd then showed some coloured slides, which are here reproduced in monochro

Slide *a* (Plate XI) shows mesenteric lymph nodes and some in the neighbourh of the pancreas; they are greatly enlarged. The largest one has been cut across you can see that there are little spots of haemorrhage and yellowish areas of necro That turned out to be a lymphosarcoma and all other nodes sectioned showed evide of the same disease, which is a systematized lymphosarcomatosis.

Slide *b* (Plate XII) shows the distribution of lymphocytes in portal areas of liver, characteristic of lymphosarcoma and also seen in lymphatic leukaemia.

Slide *c* (Plate XIII) shows the spinal cord with the posterior root ganglia dissec out on the left side of the body. The third left dorsal root ganglion is red, hav undergone haemorrhagic infarction one, of the changes one sometimes sees in se cases of herpes zoster. There was thrombosis of distended vessels in the poste root and you can see that there is intense congestion of the dorsal horns in the tra verse section of the spinal cord. This could be traced for several centimeters ab the third root.

Slide *d* (Plate XIV) shows a transverse section of the third left posterior r Thrombus distends the blood vessels and there is interstitial haemorrhage.

Slide *e* (Plate XV) this intense cuffing of the blood vessels in the posterior r of the spinal cord was found on the left hand side, only; which is the side of the b affected by the zoster inflammation. This is caused by the products of inflamma going to the spinal cord by way of the blood vessels of the left third dorsal root, so into the posterior horn.

Slide *f* (Plate XVI) shows a section of the second left posterior root ganglion wh is more or less normal. You can compare it with Slide *g* (Plate XVII) the third. I there is complete haemorrhagic necrosis of all the cells. The nuclei both of ganglion cells and of their satellite cells have disappeared and I was unable to dem strate any intranuclear inclusion bodies. In cases of zoster one expects to find t bodies fairly frequently in the satellite cells and rather rarely in the nuclei of

PLATE VII

*Herpes zoster and varicella (front).*

PLATE VIII

*Herpes zoster and varicella (back).*

[*Face page 24*

PLATE IX

*Herpes zoster and varicella (side).*

PLATE X

*X-ray of abdomen; distension of small bowel loops due to ileus.*

PLATE XI

*Mesenteric lymph nodes: lymphosarcoma.*

PLATE XII

*Liver: portal lymphocytosis due to lymphosarcoma* (× 150).

PLATE XIII

*Spinal cord; herpes zoster; haemorrhagic infarction of dorsal root ganglion of T.3 left.*

### PLATE XIV

*T.S. T.3. left posterior root; thrombosis and haemorrhage* (× 150).

### PLATE XV

*Spinal cord, posterior horn of T.3. left. Lymphocytic cuffing of vessels* (× 150).

### PLATE XVI

*T.2. left posterior root ganglion; normal* (× 150).

### PLATE XVII

*T.3. left posterior root ganglion; haemorrhagic infarction due to zoster* (× 150).

;anglion cells. Inclusion bodies may be found in the cells of a variety of different ›rgans in chicken-pox: skin vesicles, liver, lung and other places as well. In one of hese ganglion cells I found a hyaline body surrounded by a clear halo that might ıave done for an inclusion body, but it wasn't right. The halo around the inclusion ›odies should be within the nuclear membrane, and here there is so much necrosis hat none of the nuclear membranes survive.

I was disappointed in not finding inclusion bodies in the skin in this case. This nay have been due to technical troubles, or it may have been because the disease vas already in a rather late stage.

In searching for these lesions I was going very much by the descriptions of cases ›f chicken-pox by Cheatham et al. (1956). One of their cases had zoster as well as :hicken-pox and in that case they demonstrated inclusion bodies commonly in the atellite cells but rarely in the ganglion cells. One of their cases was a child with a ıeuroblastoma, and these diligent people searched through the neuroblastoma cells o find if it also was suffering from chicken-pox and they found that it was.

To sum up, this is the case of a man with lymphosarcoma who developed chicken- ›ox and herpes zoster, he went on to develop signs of intestinal obstruction and Mr. Monks was able to find a severely dilated intestine and let that down, but there was ıo actual mechanical obstruction. It was probably due to interference with the ıeuro-muscular mechanism of the bowel by the presence of the tumour he found. do not know how this works but I think he will tell us that it does happen in a ıumber of cases of paralytic ileus.

*Question:* Could you tell us if there were any vesicles in the lungs? I believe it loes occur in advanced cases of chicken-pox.

*Dr. Lloyd:* No, I did not find any. Inclusion bodies in the lung tissue have been lescribed.

*Dr. Cayton:* May I reinforce what Dr. Warin said about the importance of ex- luding smallpox where any suspicion may arise. I would like to draw your attention o the account of an epidemic of smallpox in Tottenham in 1957 (Hogben et al. 1958) n which a child, the grandson of a laboratory cleaner, dying of smallpox, led to the ecognition of the real cause of the grandmother's ill health, and to the ultimate recog- ition of an undiagnosed fatal case of haemorrhagic smallpox which came to necropsy s "acute leukaemia". Laboratory confirmation of the disease can usually be obtained vithin 18 hours of the receipt of the specimen. In smallpox relatively large inclusion odies can be seen in smears of fluid from the vesicles. I would urge that these cases re not the obvious ones and it is much better to ask for advice and get bacteriology r virology done rather than be wise after the event.

Another point: that of gamma globulin in contacts in the early stages of pregnancy. There is no direct evidence as far as I know that chicken-pox is likely to affect the oetus. Gamma globulin is very expensive and is normally only available for immedi- te contacts of german measles. We have had quite a lot now, three or four rubella ontacts every week for the last ten to fifteen weeks, and an analysis of 1700 contacts howed an attack rate of 3 per cent in those protected. When rubella is diagnosed it ; then a question of whether or not pregnancy should be terminated.

*Professor Neale:* In long past years, when liquor arsenicalis was used medically more ften for all sorts of disorders, it was noted that herpes zoster seemed to be precipi- ated by the drug. Arsenic as such is now very rarely used, but recently a new factor as been introduced. I refer to cortisone. If a person on cortisone treatment for omething else gets chicken-pox the virus might take on exceptional invasive action 1 various tissues, especially the brain, liver, lung and heart muscle, with necrotizing ffects and fatal cases have been reported. (Nicholas 1957, Haggerty & Eley 1956, .M.J. 1957, Good et al. 1957). It is simply an example that medicinal cortisone an break down the normal reactions and resistance of the individual to the virus. n fact, it may predispose the patient to a generalized spread of the virus disease and fulminating course.

You said that the rash was "the same as smallpox". I know you did not mea[illegible] but how did you come to the conclusion, at the bedside in five minutes, that it [illegible] not smallpox?

*Dr. Warin:* I do not think I said "the same as smallpox". However, judged by [illegible] rash alone the actual vesicles in this patient would have been pretty well imposs[illegible] to tell apart from smallpox, but diagnosis was easy because of the history, bec[illegible] of the big patch running round the side of the chest and because of the pain on o[illegible] But looking at one particular patch of the eruption I think it would have been [illegible] possible.

Dr. Lloyd said that the enlarged lymph nodes in the left axilla were due to secon[illegible] infection. I think we might remind ourselves that you get quite marked lymph [illegible] enlargement with herpes zoster and with herpes simplex, uncomplicated by secon[illegible] infection.

*Dr. Cayton:* In the missed case reported the primary cause of death was assu[illegible] to be acute leukaemia and it is these acutely ill patients with haemorrhagic ra[illegible] which may be confused with smallpox.

*Professor Neale:* What is the nature of the lesion usually to be found in the poste[illegible] root ganglion in cases of herpes zoster?

*Dr. Lloyd:* Usually there is a lymphocytic infiltration with congestion and oede[illegible] Inclusion bodies are to be found in satellite cells. But in quite a number of [illegible] the ganglion does undergo haemorrhagic necrosis and that was so in this case.

*Professor Neale:* Do you think it might be related to the post-herpetic neura[illegible]

*Dr. Lloyd:* I would not be surprised. Perhaps Dr. Norman could tell us?

*Dr. Norman:* I have never heard of any connection between the two.

### REFERENCES

Annotation (1957), "Dangers of chicken-pox", *Brit. Med. Jour.*, 1957, i: 511–2.

Cheatham, W. J., Weller, T. H., Dolan, T. F. and Dower, J. C. (1956) "Varicella: [illegible] of two fatal cases with necropsy, virus isolation and serologic studies," *Am. J. Path.*, **32**: [illegible] 1053.

Good, R. A., Vermier, R. L. and Smith, R. T. (1957), "Serious untoward reactions to th[illegible] with cortisone and adrenocorticotropin in pediatric practice", *Pediatrics*, **19**: 95–118 and 27[illegible]

Haggerty, R. J. and Eley, R. C. (1956), "Varicella and Cortisone", *Pediatrics*, **18**: 16[illegible]

Hewitt, M. (1954), "Herpes zoster generalisatus", *Med. Jour. of the South West,*, **70**: 5[illegible]

Hogben, G. H., McKendrick, G. D. W. and Nicol, C. G. (1958), "Smallpox in Totten[illegible] 1957", *The Lancet*, i : 1061–4 (17th May).

Nicholas, W. W. (1957), "Experiences with chicken-pox in patients receiving corti[illegible] *Amer. J. Dis. Child.*, **94** : 219.

## Figures and Tables

**Figure f1:**
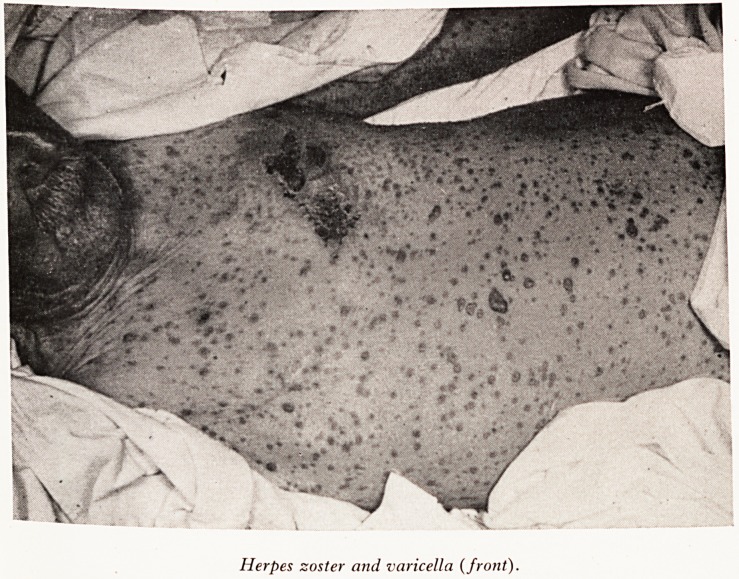


**Figure f2:**
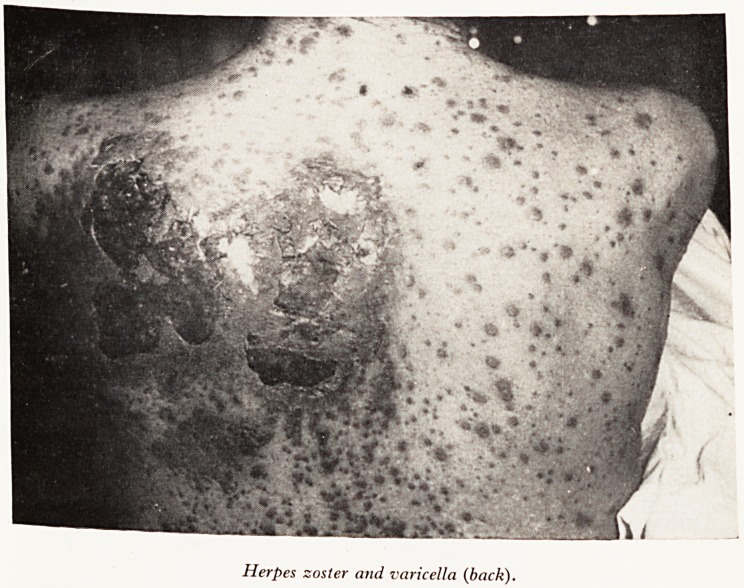


**PLATE IX f3:**
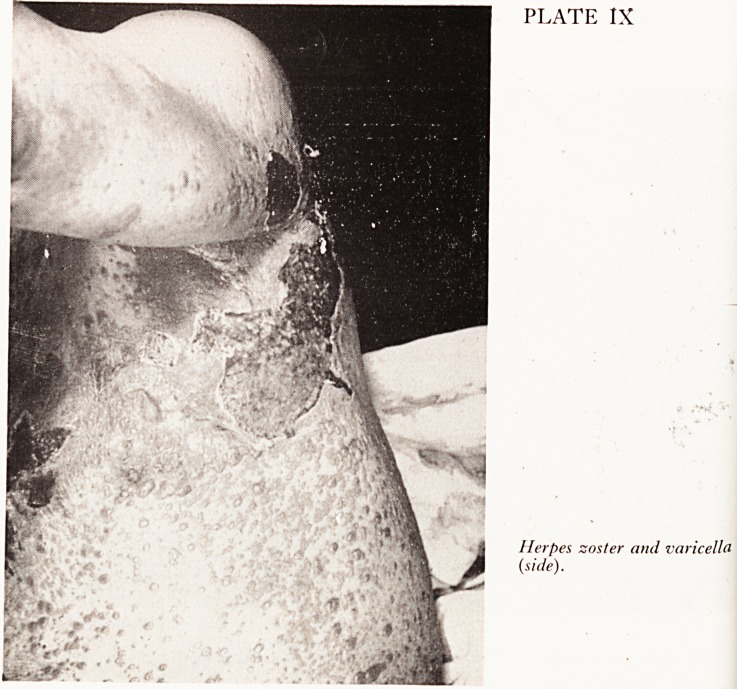


**PLATE X f4:**
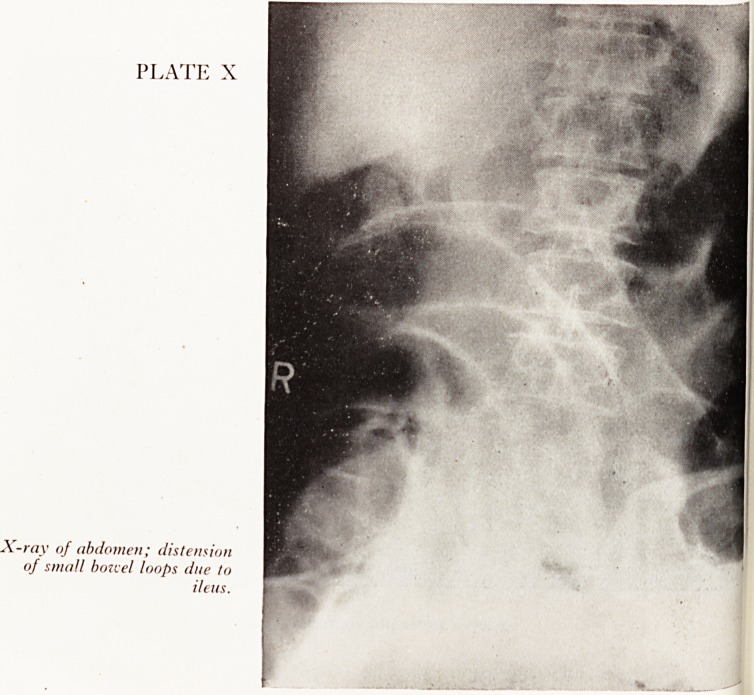


**Figure f5:**
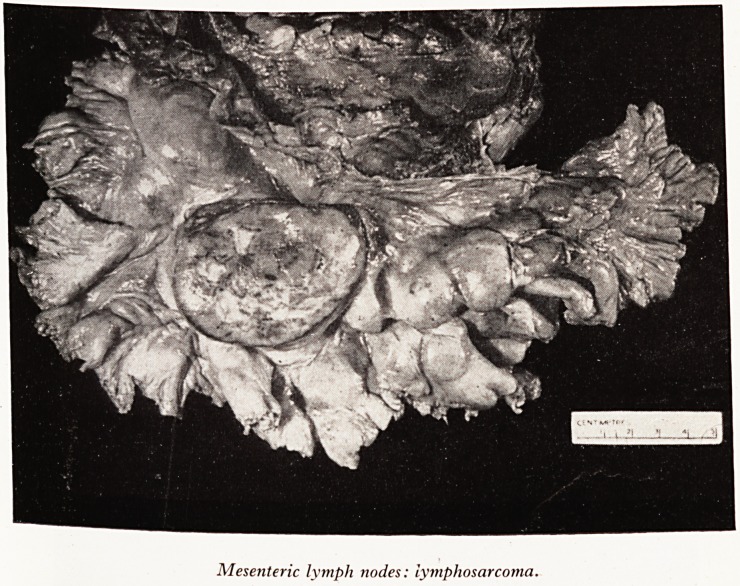


**Figure f6:**
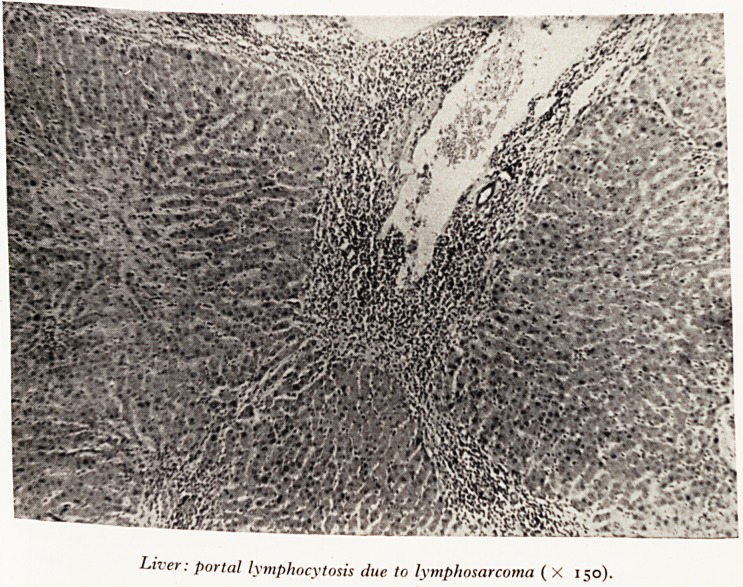


**Figure f7:**
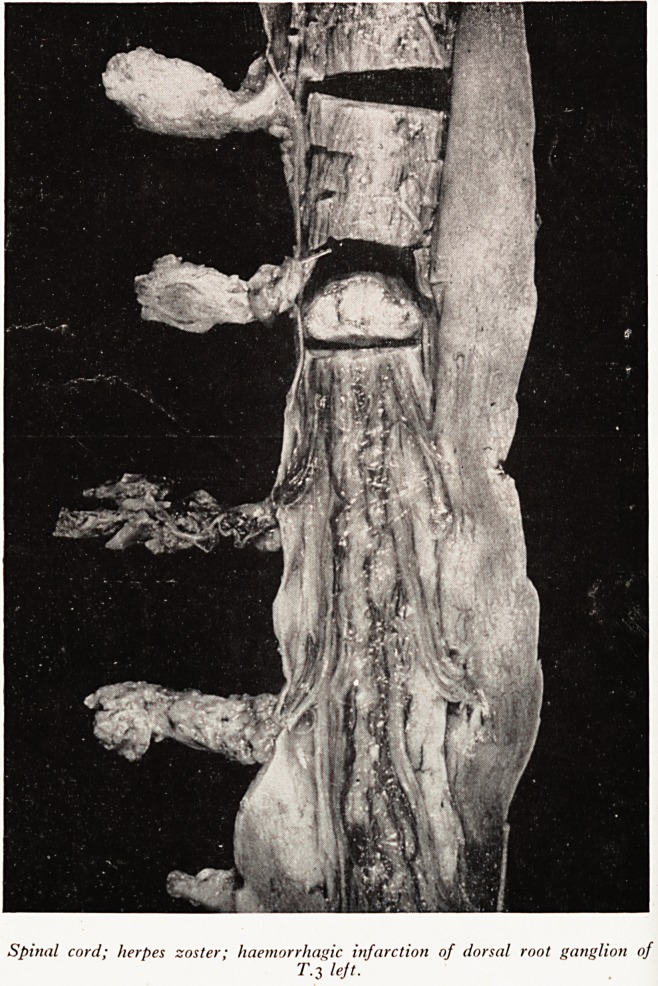


**Figure f8:**
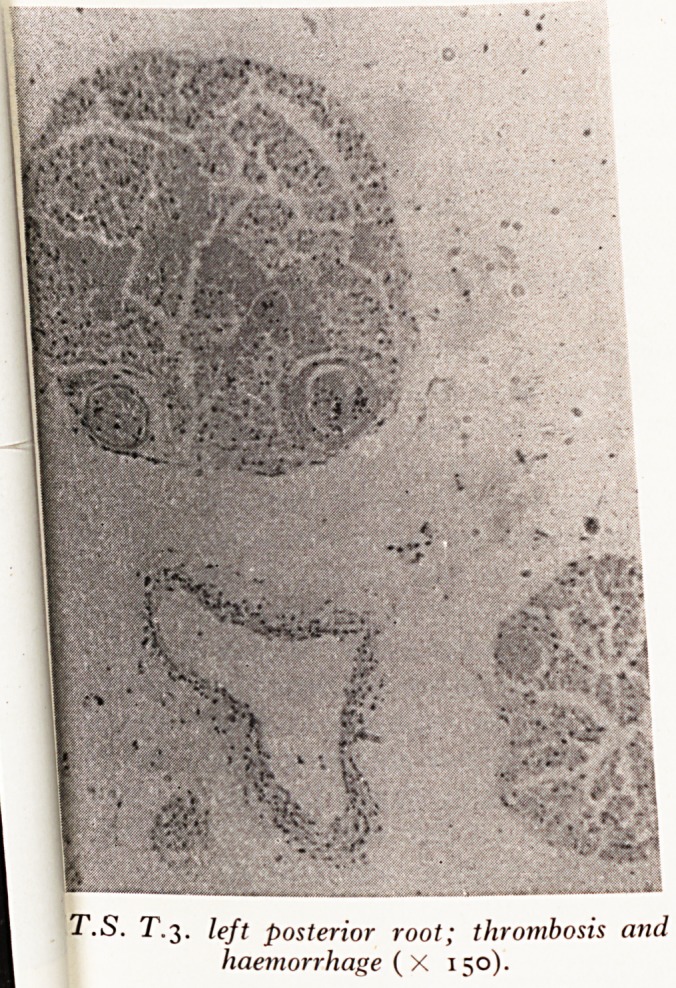


**Figure f9:**
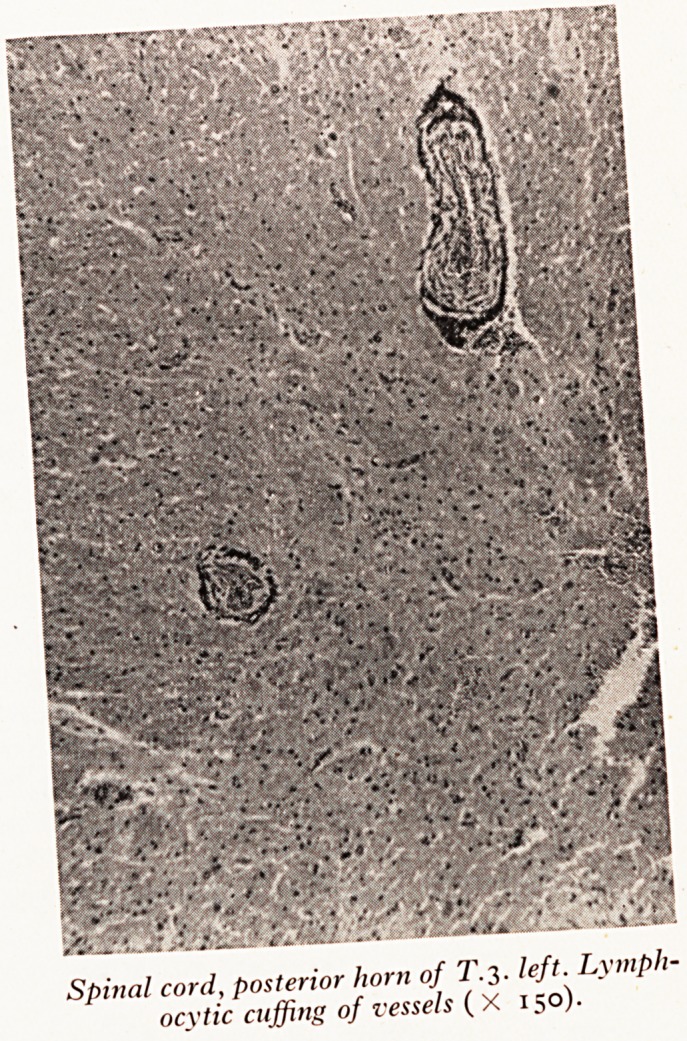


**Figure f10:**
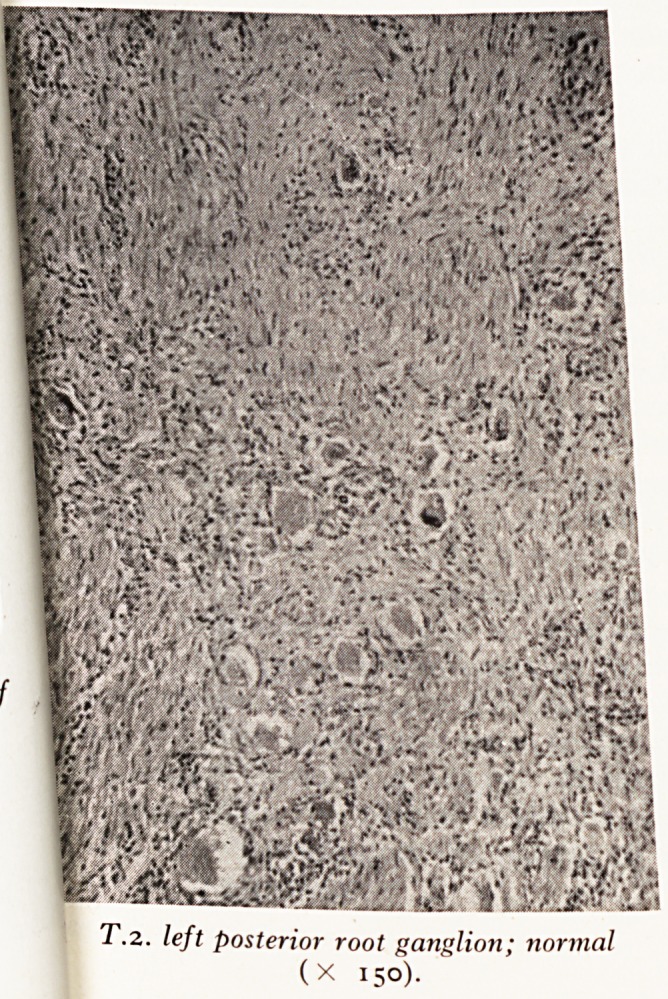


**Figure f11:**